# Predictors of overall survival in metastatic castration-resistant prostate cancer patients receiving [^177^Lu]Lu-PSMA-617 radioligand therapy

**DOI:** 10.18632/oncotarget.21600

**Published:** 2017-10-07

**Authors:** Hojjat Ahmadzadehfar, Stephan Schlolaut, Rolf Fimmers, Anna Yordanova, Stefan Hirzebruch, Carl Schlenkhoff, Florian C. Gaertner, Zool Hilmi Awang, Stefan Hauser, Markus Essler

**Affiliations:** ^1^ Department of Nuclear Medicine, University Hospital Bonn, Bonn, Germany; ^2^ Institute for Medical Biometry, Informatics and Epidemiology, University of Bonn, Germany; ^3^ Department of Urology, University Hospital Bonn, Bonn, Germany

**Keywords:** ^177^Lu, overall survival, PSMA, radioligand therapy, prostate cancer

## Abstract

Prostate-specific membrane antigen (PSMA) is a promising target for the diagnosis of and therapy for metastatic castration-resistant prostate cancer (mCRPC). The aim of this study was to measure overall-survival (OS) in mCRPC patients who received either abiraterone or enzalutamide prior to PSMA therapy. The second aim of this study was to analyse the predictors of OS according to different pre-therapeutic parameters and also the responses to the first cycle of radioligand therapy (RLT) base on PSA level. Patients with mCRPC and a history of therapy with either abiraterone or enzalutamide or both, were included in this study. Different laboratory tests and pre-therapeutic parameters have been included into the analysis. One-hundred patients received a total of 347 cycles of Lu-PSMA (median: three cycles). 69 patients showed a decline in PSA two months after the first cycle, and 38 of those patients showed a PSA decline of = > 50%. The median OS was 60 weeks. In the multivariate analysis, the level of albumin, AST and haemoglobin, existence of liver metastases and a decline of > 14% in PSA level had a significant impact on overall-survival. The median OS is significantly longer in patients without hepatic involvement, with high levels of albumin and Hb and low levels of AST. A decline in PSA levels of more than 14% was the most important response parameter with regard to overall survival.

## INTRODUCTION

Prostate-specific membrane antigen (PSMA) is a promising target for the diagnosis and therapy of metastatic castration-resistant prostate cancer (mCRPC) [1–5]. Several studies have demonstrated the safety and low toxicity profile of [^177^Lu] Lu-PSMA-617 (Lu-PSMA) for the therapy of mCRPC patients [6–12]. Prolongation of overall survival (OS) is one the most important parameters in the evaluation of efficacy of a therapeutic agent. Although, there are still no prospective studies on the OS of mCRPC patients who have undergone Lu-PSMA therapy, results from the first retrospective studies were very promising. These studies showed a positive effect of Lu-PSMA therapy on OS in patients in advanced stages of mCRPC [13–15]. Abiraterone and enzalutamide are both the approved hormone therapies for mCRPC patients [16–18]. Papers published to date on OS in mCRPC patients treated with Lu-PSMA therapy included small numbers of patients and inhomogeneous patient groups. The primary aim of this study was to measure OS in mCRPC patients who received at least abiraterone or enzalutamide as antitumor therapy prior to radioligand therapy (RLT). The second aim of this study was to analyse the predictive value of different pre-therapeutic parameters and the prostate-specific antigen (PSA) level response to the first cycle on OS.

## RESULTS

### Patients

Between December 2014 and March 2017, 150 patients were treated in our department. Fifty of these patients were excluded from the study, on the basis of the inclusion criteria, for the following reasons: 2 patients had renal cell carcinoma, 23 patients were not treated with either abiraterone or enzalutamide, 10 patients were still under therapy and had undergone less than three cycles of RLT, 3 patients had less than two months of follow-up after the last RLT cycle and 12 patients had incomplete documentation about follow-ups.

In total, 100 patients received a total of 347 cycles of Lu-PSMA (median: three cycles; range: one to eight cycles). Seventy-seven patients (77%) had a good Eastern Cooperative Oncology Group (ECOG) performance status scores (0 or 1), 22 patients had an ECOG score of 2, and one patient had an ECOG of 3. Thirty patients had a Gleason score ≤ 7, 61 had a score > 7 and the score was unknown in nine patients (Tables 1–3). At the time of analysis, 52 patients (52%) were still alive.

**Table 1:** Blood, renal and hepatic parameters prior to the first cycle of treatment

Parameter	min	max	mean	median
**Blood parameters**				
WBC (G/l) (norm: 3.6–10.5)	1.52	12.2	6.1	5.7
Hb (g/dl) (norm: 12.5–17.2)	6.0	13.6	10.9	11.3
Plt (G/l) (norm: 160–370)	62.0	562.0	248.0	240.0
**Renal parameter**				
Creatinine (mg/dl) (norm: 0.6–1.3)	0.38	1.9	0.9	0.9
**Liver function tests**				
Bilirubin total (mg/dl) (norm: 0.2–1.0)	0.2	1.7	0.4	0.4
ALT (U/l) (norm: < 50)	7.0	423	26.1	18.0
AST(U/l) (norm: < 50)	9.0	359.0.0	33.8	23.0
GGT (U/l) (norm: < 55)	15.0	808.0	65.3	35.0
Albumin (g/l) (norm: 35–52)	25.8	47.1	39.9	38.9

^*^ Sixteen patients had received a blood transfusion because of grade 3 tumour anaemia between 0 and 300 days prior to the first cycle of Lu-PSMA therapy (median: 17 days).

Abbreviations: ALT, alanine transaminase; AST, aspartate transaminase; GGT, gamma-glutamyl transferase; Hb, haemoglobin; Plt, platelets; WBC, white blood cells

**Table 2:** Tumour parameters

Parameters	min	max	mean	median
ALP (U/l) (norm: 34-117)	36	1631	250	133
LDH (U/l) (norm: < 248)	105	1875	342	250
CRP(mg/l) (norm: < 3)	0.2	166	21.8	7
PSA (ng/ml)	4.73	5910	520	206

Abbreviations: ALP, alkaline phosphatase; CRP, C-reactive protein; LDH, lactate dehydrogenase; PSA, prostate-specific antigen

**Table 3:** Patient characteristics

Gleason score			Number of patients	
6			9 (9%)	
7			21 (21%)	
8			24 (24%)	
9			31 (31%)	
10			6 (6%)	
unknown			9 (9%)	
**Prior and ongoing therapies**				
**Therapy**		**History: *n* (%)**	**Ongoing: *n* (%)**	
Prostatectomy		52 (52%)		
Abiraterone		51 (51%)	21 (21%)	
Enzalutamide		33 (33%)	39 (39%)	
Chemotherapy^*^		70 (70 %)		
Bisphosphonate or RANKL+ inhibitor		12 (12%)	69 (69%)	
Ra-223		36 (36 %)		
Regular need for analgesics!			39 (39 %)	
**Extent of disease in 100 patients, detected by 68Ga-PSMA-PET/CT**				
	**Number of patients (%)**		**Extent**	
Local recurrence	40 (40%)			
Bone metastases	98 (98 %)		< 20 metastases in 29 patients (29.6%) > 20 metastases in 45 patients (45.9%) diffuse metastases in 24 patients (24.5%)	
Lymph node metastases	78 (78 %)		iliac and abdominal in 33 patients (42.3%) thoracic in 9 patients (11.5%) iliac to thoracic in 36 patients (46.2%)	
Liver metastases	13 (13%)		singular metastasis in 1 patient multiple metastases in 12 patients	
Lung metastases	15 (15%)		singular metastasis in 2 patients multiple metastases in 13 patient	

^*^49 patients had a first-line chemotherapy treatment with docetaxel only, and 21 patients were given both docetaxel and cabazitaxel.

+ Receptor activator of nuclear factor kappa-B ligand.

! Out of 35 patients who have a regular intake of opioids

### Number of cycles

There were three patients underwent one cycle of therapy, and 18 patients underwent two cycles of therapy. One of the three patients who received one cycle of therapy was the patient who had an ECOG score of 3. This patient with diffuse bone and multiple partially PSMA negative liver metastases died 2.5 months after the first treatment cycle. The other two patients with one cycle of therapy avoided additional treatment cycles and died 16 and 18 weeks after RLT, respectively. Of 18 patients who had two treatment cycles, three showed a near complete response to the first two cycles on the basis of ^68^Ga-PSMA PET (positron emission tomography) imaging and a decline in their PSA levels to under 4 ng/ml; therefore, we decided to follow-up on these patients. The remaining 14 patients who received two cycles of treatment experienced a significant rise in PSA levels concomitant with a worsening of their general health condition, particularly with an increase in fatigue, so we decided to end the RLT. Other patients underwent at least three cycles of RLT. In this study, [3, 4, 9, 18] and 2 patients received [4, 5, 6, 7] and 8 cycles of therapy, respectively. The median injected activity per cycle was 6.0 GBq.

Sixty-two, 16 and 1 patients received [3, 4] and [5] consecutive cycles of treatment at 6- to 8-week intervals. Twenty-four patients underwent further cycles at a later time because of recurrence, which was identified based on a rise in PSA levels and disease progression that was observed in PSMA PET imaging. All of these 24 patients were patients with favourable response to the first cycle of PSMA therapies according to changes in the PSA levels.

### Treatment response to the first cycle

Sixty-nine patients (69%) showed a PSA decline 2 months after the first cycle, 38 (38%) of which showed a PSA decline of ≥ 50%.

### Survival analysis and prognostic factors

Survival was calculated from the day of the first RLT cycle. The median OS was 60 weeks in all patients, irrespective of response to treatment (95% CI: 47.3–72.7). The univariate analysis showed that low levels of aspartate transaminase (AST ≤ 24 U/l), gamma-glutamyl transferase (GGT ≤ 31 U/l), CRP ≤ 16 mg/l, lactate dehydrogenase (LDH < 225 U/l) and ALP ≤ 140 U/l; high pre-therapeutic haemoglobin (Hb ≥ 10.4 g/dl); high albumin (albumin ≥ 38.6 g/l); lower number of bone metastases; absence of liver metastases; an ECOG status of 0/1; no history of prior blood transfusion; no regular intake of analgesics; any decline in PSA levels and a decline in PSA levels of ≥ 50% were significant predictors of OS (Table 4). Analyzing the percentage of PSA decline after the first cycle determined a PSA decline ≥ 14 % as best cut-off point with a median OS of 88 weeks vs. 29 weeks.

**Table 4:** Univariate and multivariate analyses of different pre-therapeutic parameters and their impact on overall survival (only the significant parameters are shown)

Parameters	*p*-value	Median OS reported in weeks (95 % CI)	Multivariate analysis *p*-value
Number of bone metastases 29 patients ≤ 20 met 45 patients > 20 met 24 patients : super scan or diffuse bone/bone marrow met	**0.02**	94 (60.7–127.3) 55 (38.8–71.2) 37 (12.7–61.3)	ns
Existence of liver metastases 13 patients: yes 87 patients: no	**0.04**	28 (18.6–37.4) 60 (47.3–72.7)	**0.02** **HR: 3.2 (95% CI: 1.5–7.1)**
ECOG performance status (0/1 vs 2/3) 77patients: 0–1 23 patients: 2–3	**< 0.0001**	71 (43.7–98.3) 33 (24.7–41.3)	ns
Blood transfusion prior to the first cycle 16 patients: yes 84 patients: no	**0.04**	36 (28.3–43.7) 63 (49.5–76.5)	ns
Albumin (cut-off: 38.6 g/l) 49 patients ≤ 38.6 51 patients > 38.6	**0.0003**	38 (26.0–50.0) 95 (47.3–142.7)	**0.01** **HR: 0.9 (95% CI: 0.8 – 0.95)**
AST (cut-off: 24 U/l)) 56 patients ≤ 24 44 patients > 24	**0.0002**	88 (61.1–114.9) 36 (16.8–55.2)	**0.04** **HR: 2.5 (95% CI: 1.3 –4.8)**
GGT (cut-off: 31 U/l) 42 patients ≤ 31 58 patients > 31	**0.0001**	88 (63.4–112.6) 47 (31.5–62.5)	ns
ALP (cut-off: 140 U/l) 52 patients ≤ 140 48 patients > 140	**0.0003**	71 (45.9–96.0) 42 (24.0–60.0)	ns
ALP (cut-off: 220 U/l) 65 patients ≤ 220 35 patients > 220	**0.019**	70 (56.9–83.1) 37 (17.7–56.2)	ns
LDH (cut-off: 225 U/l) 36 patients < 225 64 patients ≥ 225	**< 0.0001**	not reached 46 (29.6–62.4)	ns
Hb (cut-off: 10.4 g/dl) 33 patients <10.4 67 patients ≥10.4	**0.0001**	36 (24.5–47.5) 88 (60.7–115.3)	**< 0.0001** **HR: 0.5 (95% CI: 0.2–0.9)**
CRP (cut-off: 16 mg/l) 65 patients ≤ 16 35 patients > 16	**0.0001**	71 (46.8–95.2) 33 (25.2–40.8)	ns
Regular need for analgesics 39 patients: yes 61 patients: no	**0.007**	36 (24.7–47.3) 70 (56.1–83.9)	ns
Regular need for opioids 35 patients: yes 65 patients: no	**0.0002**	33 ( 24.9–41.1) 88 (48.0–127.9)	ns
PSA percent change (cut-off: –14%) 62 patients: decline > 14 % 38: no PSA decline or decline ≤ 14%	**< 0.0001**	88 (58.3–117.7) 29 (20.1–37.8)	**< 0.0001** **HR: 6.9 (95% CI: 3.6 - 13.3)**
PSA any decline 69 patients: yes 31 patients: no	**< 0.0001**	71 (45.4–96.6) 29 (20.5–37.5)	ns
PSA decline ≥ 50% 38 patients: yes 62 patients: no	**0.009**	70 (39.5–100.5) 49 (30.2–67.8)	ns

Abbreviations: ALP, alkaline phosphatase; ALT, alanine transaminase; AST, aspartate transaminase; CRP, C-reactive protein; GGT, gamma-glutamyl transferase; Hb, haemoglobin; HR, hazard ratio; LDH, lactate dehydrogenase; ns, not significant; Plt, platelets; PSA, prostate-specific antigen; WBC, white blood cells.

**Figure 1:**
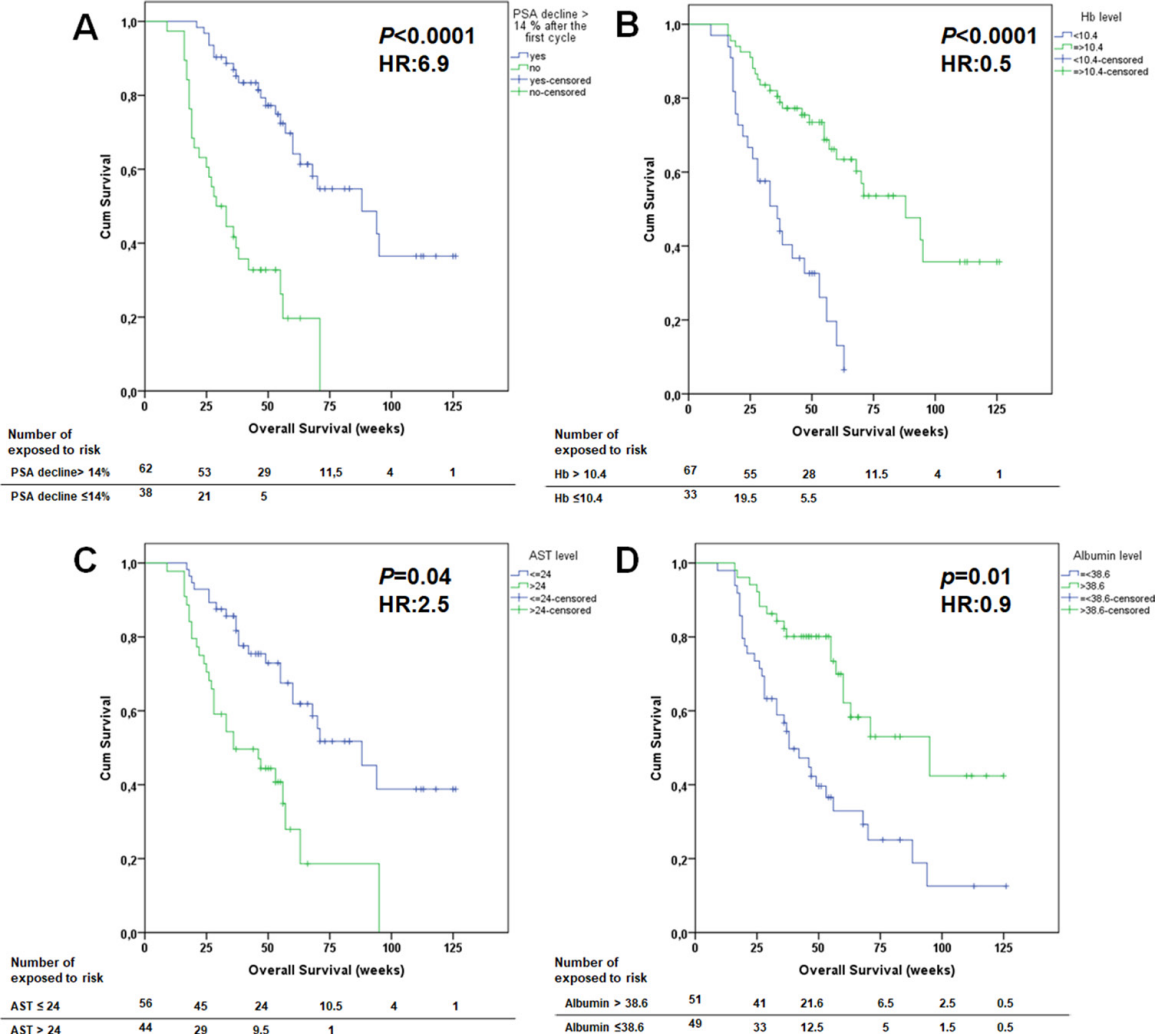
Kaplan-Meier survival curves of prostate cancer patients treated with Lu-PSMA stratified by various prognostic variables in the multivariate analysis (**A**) a prostate-specific antigen (PSA) decline more than 14%. (**B**) Hb level prior to the first cycle. (**C**) AST level prior to the first cycle. (**D**) albumin level prior to the first cycle.

In the multivariate analysis, only albumin, AST and Hb levels, existence of liver metastases and a PSA decline ≥ 14% remained significant (Table 4) (Figure 1). The other pre-therapeutic parameters were not significant with regard to OS.

**Figure 2:**
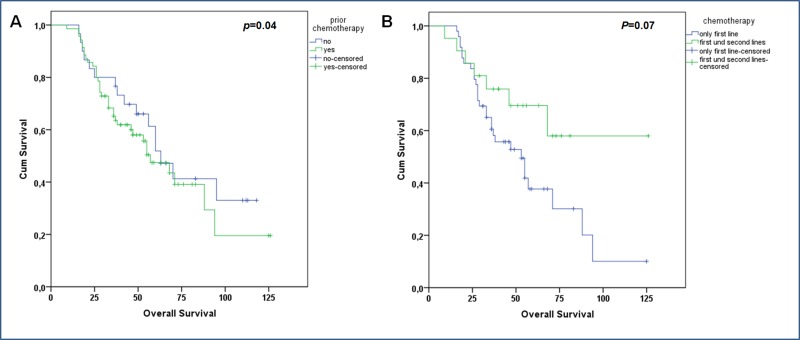
Kaplan-Meier survival curves of patients according to prior chemotherapy (**A**) no impact of prior chemotherapy on overall survival. (**B**) Overall survival considering the number of lines of chemotherapy. Although patients with the history of both docetaxel and cabazitaxel show longer median overall survival, this difference was not significant.

Prior chemotherapy was not a significant predictor of OS. In the current study, 70 patients had been treated previously with chemotherapy; of those, 21 patients also had second-line chemotherapy with cabazitaxel. The patients with prior chemotherapy had a median OS of 57 weeks (90% CI: 40.2–73.8) compared to 63 weeks (95% CI: 49.1–76.9) in patients without chemotherapy (*p* = 0.40) (Figure 2A).

Forty-nine patients received only docetaxel; those patients had a median OS of 53 weeks (90% CI: 34.5–71.5). The median OS in the 21 patients underwent both docetaxel and cabazitaxel had not yet been reached (*p* = 0.07) (Figure 2B).

## DISCUSSION

According to several retrospective studies, there is a high response rate to Lu-PSMA therapy [6–9, 19, 20] and a very low probability of high-grade hematotoxicity [8, 12] or nephrotoxicity [8, 11, 21]. However, apart from a good response and a low-toxicity profile, prolongation of overall survival is the most important factor for the evaluation of the efficacy of a new therapeutic agent.

Rahbar et al. [14] compared 28 patients who were treated with a total of 50 cycles of RLT (one to two cycles per patient) with a historical patient cohort treated with the best supportive care prior to the availability of Lu-PSMA [14]. The estimated median OS in their study was 29.4 weeks, which was significantly longer than the OS of 19.7 weeks (*p* = 0.031) that was observed in the historical best supportive care group. Rahbar et al. did not compare the median OS between responders and non-responders, and the majority of patients in this study underwent further cycles of Lu-PSMA therapy after the data had been published.

In a recently published study from our group, 52 patients who received a total of 190 cycles of RLT (three to six cycles per patient) were analysed. In 80.8% of patients, a decline in PSA levels two months after the first cycle was observed, with 44.2% showing a PSA decline of ≥ 50%. The median OS was 60 weeks for all patients. The median OS was significantly longer for patients who showed any PSA decline after the first cycle compared to patients without a PSA decline (68 vs 33 weeks). These results were very promising because they showed that Lu-PSMA induces a good response, and in responders it induces a significantly longer OS. In that study, a majority of patients had a history of therapy with abiraterone and/or enzalutamide, and 55.8% and 44.0% of patients received chemotherapy and a therapy with Ra-223, respectively.

Bräuer et al. [13] confirmed the results of our published study. They investigated a group of 59 patients who underwent a total of 159 cycles of RLT (median: three cycles). They reported that patients with any decline in PSA levels in response to the first cycle of RLT had a significantly longer median OS (56 weeks) compared to OS of patients with PSA progression (29 weeks). All of the patients in their study had had at least abiraterone or enzalutamide; 80% and 10% of the patients had been given chemotherapy and/or therapy with ^223^Ra, respectively.

In both studies [13, 15], patients with a decline in PSA levels of ≥ 50% after the first RLT did not have a significantly longer OS than those with a PSA decline of < 50%. Both studies were limited by a small number of patients, and there was a need to investigate these results in a larger cohort.

Currently, abiraterone and enzalutamide are among the first line of therapies used to treat mCRPC [18]. In this study patients who received at least one of these therapies were included. Of the 100 patients included in the current study, 70% had been given at least a first line chemotherapy with docetaxel. 36% had been given ^223^Ra therapy. All of the patients suffered from very advanced disease, with 98% having bone involvement, 70% more than 20 lesions up to super scan, and13% having hepatic metastases.

Despite their advanced disease and history of various therapies, the median OS was 60 weeks in all patients. The median OS of patients in the study of Bräuer et al. [13] was 32 weeks, which was shorter than the median OS of the patients in our study. This may be because of 48% of the patients in their study had an ECOG of 2 or 3 compared to only 23 % of the patients in our study. In the current study, patients with an ECOG of 0 or 1 showed significantly longer median OS compared to patients with an ECOG of 2/3 (77 weeks vs. 33 weeks; *p* < 0.0001). Ferdinandus et al. evaluated the prognostic value of various pre-therapeutic parameters on therapy response, based on changes in PSA after the first cycle of RLT [22]. Their multivariate analysis of these parameters showed that patients with a high platelet count or a regular need for analgesics had a significantly worse response to the first RLT cycle, considering any PSA decline after two months. When a PSA decline of ≥ 50% was considered, patients with a regular need for analgesics showed a worse response in the multivariate analysis; other pre-therapeutic parameters had no impact on the response to RLT [22].

Based on the study of Ferdinandus et al., we evaluated the impact of numerous pre-therapeutic parameters on OS (Tables 1–3). In the univariate analysis, some of these parameters as well as response to the RLT as measured by PSA were significant (Table 4). In contrast to previously published results from our team and the University Hospital Muenster [13, 15], patients with a decline in PSA levels of ≥ 50 % showed a significantly longer median OS (70 weeks; 95% CI: 39.5–100.5) than those patients with decline in PSA of < 50 % (49 weeks; 95% CI: 30.2–67.8).

According to various guidelines and trials, response to a therapy is defined as a ≥ 50% decline in the PSA levels [16, 17, 23, 24]; however, patients treated with RLT are typically in a very aggressive phase of their disease with a rapidly rising PSA level prior to RLT. Thus, a PSA level that was unchanged two months after the first cycle of RLT could be considered as a response to treatment. In the current study, as with the other two studies mentioned above, patients with any decline in PSA levels showed a significantly longer OS. A decline in PSA of 14% was a significant cut-off point in both the univariate and the multivariate analysis. Patients with a PSA decline of > 14% had a median OS of 88 weeks (95% CI: 58.3–117.7) compared with 29 weeks in patients with PSA decline of ≤ 14% (95% CI: 20.1–37.8) (*p* < 0.0001).

Thirty-nine patients (39%) required analgesics regularly. The need for pain killers was a negative predictor of OS (*p* = 0.005), which was in concordance to the results of Ferdinandus et al. who reported a worse response in these patients [22]. A majority of these patients (35/39) took an opioid and had a significantly shorter OS of 33 weeks vs 88 weeks for those not on opioids (*p* = 0.0002).

The baseline level of PSA was not significant predictor of OS. However, ALP, LDH and CRP (which could be used as tumour-burden parameters) each was a significant predictor of OS in the univariate analysis. In concordance with other studies [13, 25], we found that an ALP cut-off level of 220 U/l was significant in the univariate analysis. In addition, we found that a cut-off level of 140 U/l was more significant than a cut-off level of 220 U/l (Table 4).

The Hb level was significant in both univariate and multivariate analyses (Table 4). Patients with a Hb level ≥ 10.4 g/dl had a longer OS (*p* < 0.0001) than those with Hb < 10.4 g/dl. AST, GGT and albumin each was a significant predictor of OS (Table 4).Of these parameters, AST and albumin were also significant in the multivariate analysis. The extent of bone metastases and the existence of liver metastases were significant predictors of OS, with hepatic involvement being significant in the multivariate analysis.

Prior chemotherapy did not have an impact on OS. Of the 70 patients who had received chemotherapy, the median OS in patients with a history of both docetaxel and cabazitaxel treatment was longer than patients treated with docetaxel alone, but the difference was not significant in this study. This may be because the majority of patients with a history of treatment with only docetaxel were not fit enough for therapy with cabazitaxel at the time of the RLT; however, this aspect should be evaluated in a larger cohort of patients.

The median OS in mCRPC patients receiving RLT in advanced stages of disease is 60 weeks. The median OS is significantly longer in patients without hepatic involvement, with high levels of albumin and Hb and low levels of AST. A decline in PSA levels of more than 14% measured two months after the first cycle of RLT was the most important response parameter with regard to OS.

## MATERIALS AND METHODS

All patients who underwent Lu-PSMA therapy in our department from Dec 2014 to March 2017 were evaluated for inclusion in this study. Eligible patients were selected according to following inclusion criteria: (1) patients with mCRPC and PSMA positive metastases in the PSMA imaging; (2) patients with a history of therapy with either abiraterone or enzalutamide or both; (3) patients treated with at least three cycles of RLT, or patients with 1–2 cycles of RLT who died before the third cycle, did not receive the third cycle because of significant worsening of their general condition or because of an excellent response to the first or second cycle; (4) patient follow-up of at least two months after the last cycle or the patient died; and (5) complete documentation.

### Patients

In this study, mCRPC patients with distant metastases and progressive disease (determined by PSA levels) were treated with multiple cycles of Lu-PSMA, with a median interval of eight weeks between each cycle (range: six to eight weeks). Patients were followed-up for at least two months after the last cycle. Some of the data from 45 patients who were included in this study had been previously reported [6–9, 12, 15, 22, 26–28]; however, the current study included a greater number of patients that had all received at least abiraterone or enzalutamide. Additionally, in this paper we have evaluated the predictive factors of OS in a larger cohort of patients. This retrospective study was approved by the hospital’s ethics committee. Written informed consent was provided by each patient.

### Laboratory tests

One day prior to each therapy cycle, the following markers were assessed in all patients: hematological and renal status, liver function, PSA, ALP and blood biochemistry. Only the laboratory values obtained shortly prior to the first cycle were included in the analysis (Tables 1 and 2).

### Parameters evaluated for predictive factors of OS

All the laboratory tests and parameters shown in tables 1–3 were included in the analysis. These included the extent of bone metastases, the existence of liver and lymph node metastases, age, the need for pain medication, the ECOG score and prior received cancer-specific therapies.

### Treatment

The PSMA ligand (PSMA-617) was obtained from ABX GmbH (Radeberg, Germany). The preparation of Lu-PSMA has been explained in detail in a previous publication [7]. The treatment solution was administered by slow intravenous injection over 30–60 seconds, followed by 1000 ml of NaCl or Ringer’s solution. All patients were discharged 48 hours after therapy, in accordance with the rules of the Federal Office for Radiation Protection in Germany (BfS).

### Tumour response evaluation

PSA was used as the main marker for response evaluation. Changes in the PSA level were classified as either a decrease of ≥ 50% or any percentage decrease in PSA. Any increase in PSA was considered to indicate disease progression. According to our previous study [15] a the study by Brauer et al. [13], responders to the first cycle of Lu-PSMA tend to live significantly longer than non-responders. For that reason, only the response to the first treatment cycle was considered as a possible predictive parameter in this study.

### Statistical analysis

The Log-rank test was used to compare survival times between groups of patients defined by qualitative criteria. For continuous variables Maximally Selected Rank Statistics [29] were used to derive optimal cut-off points and a statistical assessment at once. Stepwise Cox regression was used to fit a multivariable risk model to the data.
